# Spectroscopic characterization of DOM and the nitrogen removal mechanism during wastewater reclamation plant

**DOI:** 10.1371/journal.pone.0187355

**Published:** 2017-11-17

**Authors:** Lei Wang, Ying-Jun Li, Ying Xiong, Wen-Bing Tan, Lie-Yu Zhang, Xiang Li, Xiao-Shu Wang, Jian-feng Xu, Tong-Tong Li, Jin-Sheng Wang, Ming-Xuan Cai, Bei-Dou Xi, Di-Hua Wang

**Affiliations:** 1 School of Resources and Environmental Science, Wuhan University, Hubei, Wuhan, P. R. China; 2 State Key Laboratory of Environmental Criteria and Risk Assessment, State Environmental Protection Key Laboratory of Simulation and Control of Groundwater Pollution, Chinese Research Academy of Environmental Science, Beijing, China; 3 Beijing Vocational College of Agriculture, Beijing, P. R. China; 4 Key Laboratory of Urban Storm Water System and Water Environment, Ministry of Education, Beijing University of Civil Engineering and Architecture, Beijing, China; 5 College of Resource Environment and Tourism, Capital Normal University, Beijing, China; 6 College of Environmental Science and Engineering, Guilin University of Technology, Guilin, China; Universidade Estadual Paulista Julio de Mesquita Filho, BRAZIL

## Abstract

The performance of the Sha-he wastewater reclamation plant was evaluated in this study. To remove residual nitrogen after Anaerobic-Anoxic-Oxic (A^2^O) treatment, three multistage Anoxic-Oxic (A/O) were added to investigate the nitrogen removal efficiency and its mechanism. In addition, the constituents and evolution of dissolved organic matter (DOM) during wastewater reclamation was also investigated using a method combining fluorescence spectroscopy with fluorescence regional integration (FRI). The results suggested that multistage A/O treatment can effectively improve the nitrogen removal ability under low concentrations of carbon sources. The total nitrogen (TN) exhibits significantly positive correlation with fulvic acid-like materials and humic acid-like materials. The correlation coefficient for TN and fulvic acid-like substances (R^2^ = 0.810, *P* < 0.01) removal was greater than that of humic acid-like substances (R^2^ = 0.636, *P* < 0.05). The results indicate that nitrogen removal may be achieved with the fulvic-like and humic-like substances, and the removal effects were higher by fulvic acid-like substances than humic-like substances, mostly due to that the latter were relatively more difficult to be utilized as carbon source during the nitrogen removal process. The effluent water quality of biological treatment reached the first grade A standard of “Cities sewage treatment plant pollutant discharge standard” (GB18918-2002). In addition, the effluent from the membrane bioreactor reached the “Standards of reclaimed water quality” (SL368-2006).

## Introduction

In recent years, the lack of drinking water has attracted more attention in light of restricted effluent standards and the increasing population [1]. Traditionally, studies have always focused on advanced water treatment [2]. To alleviate water shortages, wastewater reclamation was introduced as an effective method for use in the water cycle [3, 4]. The reuse and recycling of wastewater have been considered as alternative resources, and they have played a prominent role in alleviating water scarcity in recent decades [5]. Wastewater reuse is currently considered as the most important element of sustainable water management globally. Because reclaimed water has many benefits, such as low cost, easy operation, environmental friendliness and sustainability, increasing countries and enterprises are adopting this technology for vehicle cleaning, toilet flushing, industrial cooling, agricultural and landscape irrigation [3, 6, 7]. However, most sewage treatment plants receive complex influents consisting of municipal and industrial mixtures, thus the broad application of reclaimed water has been limited by water quality and safety [8]. Especially, the excess nitrogen found in reclaimed water would be one of the most important factors, and emission and utilization of reclaimed water without control would cause eutrophication [9].

The key of wastewater reclamation is the removal of nitrogen, especially under low carbon source conditions, which would be helpful for meeting the demand for water [10, 11]. Previous studies have suggested that refractory organics could be translated into biodegradable organic compounds, which could be used as a carbon source during the denitrification process within deep subsurface wastewater infiltration systems [11]. Moreover, similar results were also observed when treating mature leachate from a landfill with an up-flow anaerobic sludge blanket (UASB), an anoxic/aerobic (A/O) reactor, and an anammox reactor (ANAOR), the synergism of denitrification and organic matter removal was accomplished without adding external carbon sources [12]. Therefore, to achieve effective nitrogen removal, it is urgent to find a rapid and effective method to investigate the composition and transformation of organic matter. EEMs has been used more frequently to reflect the characteristics of aromatic or unsaturated compounds and to trace the source, composition and reaction of DOM in wastewater [13].

To explore the mechanism and the relationship between nitrogen removal and DOM transformation, the DOM from a wastewater reclamation process was investigated using EEMs [14, 15]. EEMs is a rapid, sensitive and small sample-volume analysis method without sample pretreatment prior to analysis, and it is potentially suitable for the online monitoring of the reactivity and reliability of DOM [16]. However, there is insufficient knowledge about the fraction variation of organic matter that remains in the water treatment system. Nevertheless, fluorescence EEMs analysis has generally been limited to the visual identification of peaks or ratios of peak intensities [17]. These techniques lack the ability to capture the heterogeneity of samples [18]. Additionally, the composition of DOM in the aquatic environment was extremely complex, and many components were not identified. Therefore, it is essential to apply fluorescence regional integration (FRI) to the monitor water treatment systems. FRI is a new analytical approach that was proposed by Chen’s group, by which EEMs are divided into five individual regions and calculated to analyze all the wavelength-dependent fluorescence intensity data from EEMs spectra and determine the fluorescence DOM components in a quantitative manner [19, 20].

In this study, wastewater samples were collected from different units of the Sha-he wastewater reclamation plant (WRP) in Beijing, China. For each unit of the plant, the nitrogen transformation processes of wastewater reclamation were examined. In addition, the DOM transformation mechanisms were also analyzed to investigate the effects of DOM on nitrogen removal. Furthermore, the correlation between variations in the nitrogen composition and the EEMs fluorescence regional integration was also investigated.

## Materials and methods

### Experimental equipment

Sha-he town is located in the Changping district of Beijing, which is under development and construction. The water consumption in this area is far greater than the water supply, which has led to a water supply shortage. Therefore, reusing the treated municipal sewage through the Sha-he wastewater reclamation plant is effective for abating the area's extremely urgent water stress.

**Fig 1:**
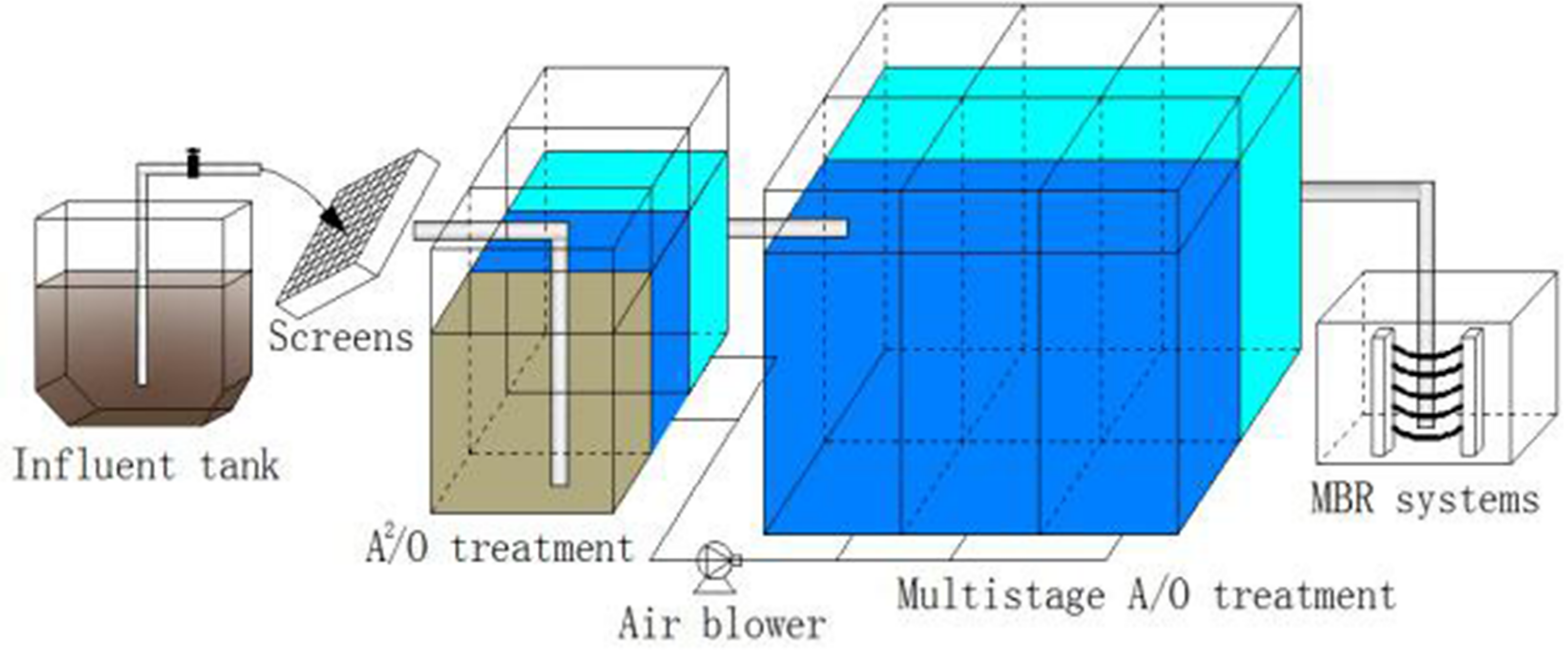
A schematic diagram.

The water samples used in this study were collected from the Sha-he wastewater reclamation plant, which is attached to Beijing Enterprises Water Group Limited Company in Beijing, China. The samples collection was conducted under the permission of the company. As shown in **Fig 1**, the Sha-he WRP consists of coarse and fine screens, biological Anaerobic-Anoxic-Oxic (A^2^O) treatment and three multistage Anoxic-Oxic (A/O) treatment, as well as a sedimentation tank and membrane bioreactor (MBR) filled with submerged hollow fiber membrane modules [21]. The capacity of the WRP is 200,000 m^3^/d, with a hydraulic retention time (HRT) of 24 h and a sludge retention time (SRT) of 30 d. The influent from the WRP was domestic wastewater with the following typical characteristics: 274.9 ± 5.7 mg L^-1^ chemical oxygen demand (COD), 60.6 ± 1.9 mg L^-1^ total nitrogen (TN) and 17 ± 0.15 mg L^-1^ total phosphorus.

### Sample collection and storage

The containers were pre-conditioned with successive ultrassive ultrapure water rinses. Water samples were collected from each unit and passed through pre-washed 0.45μm membrane filters. The water samples were maintained in a dark room at 4°C in fridge and used within 72 h.

Sample collected from influent named 1#, and sample collected after coares and fine screens named 2#. During the A^2^O treatment, the samples collected from the anaerobic period, anoxic period and aerobic period named 3#, 4# and 5#, respectively. During the multistage A/O treatment, samples collected from each A/O treatment named 6#, 7# and 8#, respectively. Sample collected in the sedimentation tank named 9#, and sample collected after MBR named 10#.

### Analytical methods

The COD was measured according to Chinese SEPA Standards (State Environmental Protection Administration, 2002). The dissolved organic carbon (DOC) concentrations of all the samples were measured according to non dispersive infrared absorption method, using an Analytik Jena Multi N/C 2100 DOC analyzer (Analytik Jena, Jena, Germany) set at approximately 15 mg L^-1^ for the fluorescence analyses. The TN, nitrate nitrogen (NO_3_^-^-N) and nitrite nitrogen (NO_2_^-^-N) were determined using ultraviolet and visible spectrophotpmetry methods, ammonia nitrogen (NH_4_^+^-N) was determined using nessler's reagent dectrophotometry method[22]. All the samples were analyzed three times, and the results are shown as the averages and standard deviations. The EEMs samples were tested using a fluorescence spectrophotometer (Hitachi F-7000, Tokyo, Japan), and they were collected on a 700-voltage xenon lamp using a scanning speed of 12,000 nm/min at laboratory temperature (25.0 ± 2°C), with excitation wavelengths varying from 200 to 450 nm in 5 nm steps and emission wavelengths from 200 to 500 nm in 5 nm steps. During the test, Raman water scatter peaks were eliminated by subtracting the EEMs of ultrapure water blank. In addition, due to the instrument component characteristics, the spectroscopy was likely to be distorted, thus a spectroscopic correction was required to eliminate the interference [20].

### Fluorescence regional integration analysis

FRI was performed according to the published literature to calculate the total and regional fluorescence intensities for each sample. This integration was based on five regions that were previously defined by Chen et al [19], with slightly modified and representative aromatic proteins (I), tyrosine-like substances (II), aromatic proteins and tryptophan-like substances (III), fulvic-like and humic-like substances, (IV) microbial byproducts, proteins, tryptophan-like substances and biopolymers and (V) humic-like substances. The fluorescence DOM components obtained through the fluorescence regional integration of regions I, II, III, IV and V were denoted as Ф_I,n_, Ф_II,n_, Ф_III,n_, Ф_IV,n_ and Ф_V,n_, respectively. The total fluorescence was calculated by integrating the volume under the whole EEM surface, which was denoted as TOT.

### Statistical analysis

Statistical analysis was carried out using the software SPSS version 19.0 for Windows (SPSS, Chicago, IL). Pearson’s correlation coefficient (R) was used to evaluate the linear correlation between two parameters. Pearson’s coefficient is always between -1 and +1, where -1 denotes a perfect negative correlation, +1 a perfect positive correlation, and 0 the absence of a relationship. The correlations were considered statistically significant at a 95% confidence interval (*p* < 0.01).

## Results and discussion

### Total removal performance

Following A^2^O and multistage A/O treatment, the effluent water quality reached the “Cities sewage treatment plant pollutant discharge standard” (GB18918-2002), which is the first grade A standard [22]. The effluent from the membrane bioreactor reached “Standards of reclaimed water quality” (SL368-2006) as published in 2007. In the wastewater reclamation plant, the effluent NH_4_^+^-N, TN, COD and DOC removal efficiencies were 99.74%, 94.54%, 95.14% and 92.93%, respectively. In particular, the multistage A/O process is the key of TN removal in the case of low concentrations but high performance. It was also observed that the multistage A/O system could effectively achieve denitrification without a carbon source supplement. It was confirmed that the multistage A/O system was a technically feasible and economically favorable dynamic process for removing nitrogen and organic composition simultaneously during wastewater reclamation.

### Organic component removal performance

#### Variations in COD and DOC

**Fig 2:**
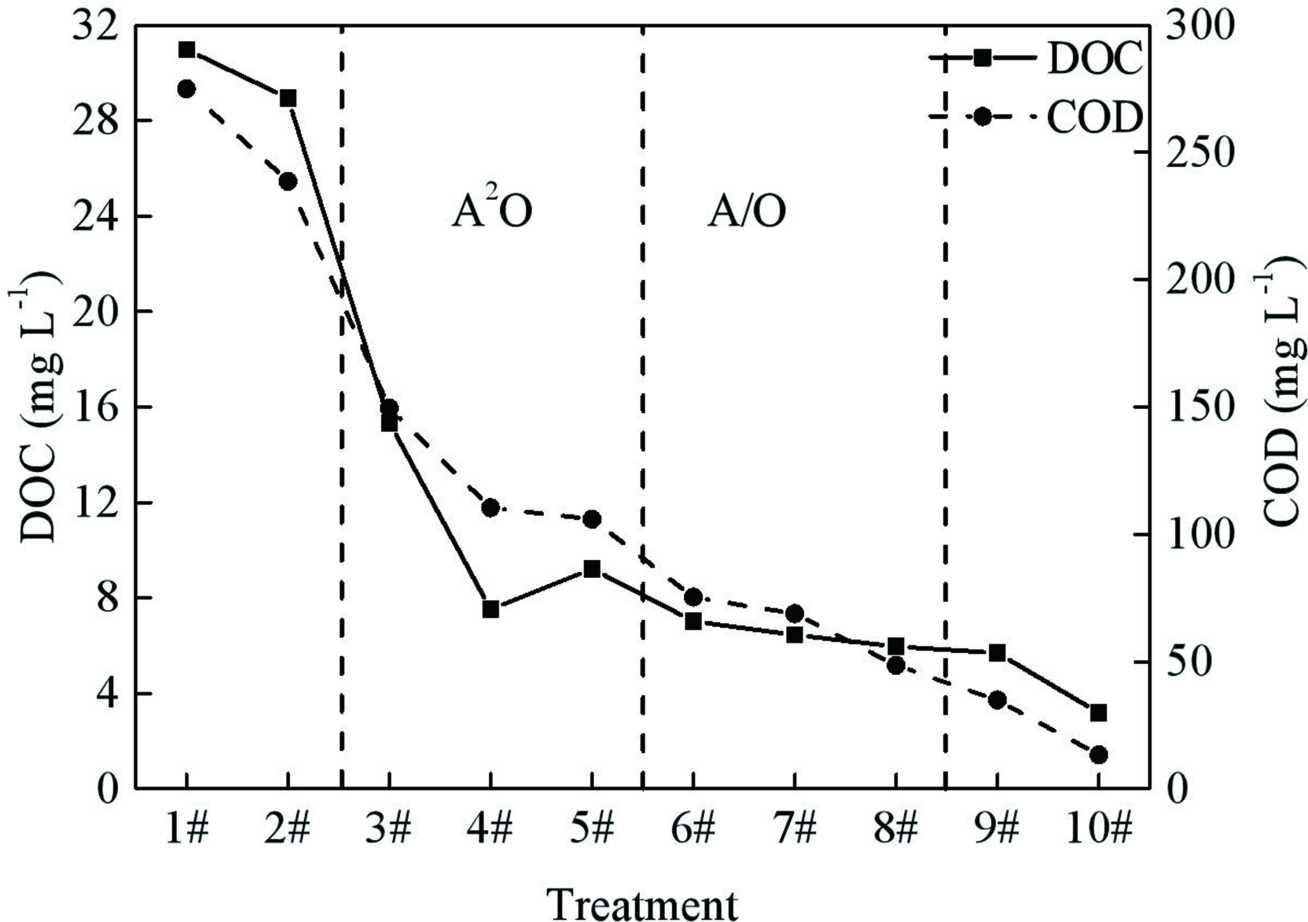
Variations in the COD and DOC.

The variations of COD and DOC in the wastewater reclamation plant are shown in **Fig 2**. The influent first flowed through the coares and fine screen, and a 13.19% nosedive of COD was noted. This decrease can be attributed to the retention of solid particle COD by the double screen. In the A^2^O portion, the maximum COD removal efficiency was observed to be 55.61% during the anaerobic period, which is consistent with earlier reports that the anaerobic period was the primary location for COD consumption [23, 24]. In this zone, macromolecular organic compounds were gradually converted into small-molecule organic compounds by hydrolysis-acidification, and these compounds be used as a carbon source for denitrification during the anoxic period [25]. This phenomenon is consistent with earlier reports in which approximately 60–70% COD could be taken up during anaerobic and anoxic period, and the low COD concentration in the supernatant during the aerobic period was suitable for nitrification [26]. Although sufficient oxygen concentrations were present during the aerobic period, the change in COD concentrations was limited. This result indicated stronger resistance to organic loading, and only the most easily degradable organic matter was degraded during the aerobic period [27, 28]. The COD concentrations decreased by 54.16% in the multistage A/O section, which could further improve the COD removal. Finally, the full removal of COD was achieved by the membrane, which was helpful for trapping the non-biodegraded particulate COD as well as a portion of the soluble COD (in a range from 0.04–0.45 μm) [29]. High removal efficiencies for COD seem to be one of the major advantages of MBR systems [30, 31]. Molecules in the MBR effluent were filtered by porosity structure, so the COD in the water was close to 0 mg L^-1^.

Moreover, the present study frequently suggested a correlation between the DOC content and the COD concentrations [32]. A correlation analysis showed that the COD and the DOC in this system were significant and positive, whereas the DOC in the effluent of each unit was still considerable. The DOC value has been regarded by a number of researchers as a significant parameter that could reflect information about its sources and transformation products in relation to treatment and human behavior [33–35]. Jaffé et al reported that the DOC concentration reflected the amount of DOM [36]. DOC was considered the most comprehensive surrogate parameter for quantifying the DOM concentrations, so the utilization of DOM should not be neglected [37, 38]. The results showed that the multistage A/O process contributed more in the DOM removal. In particular, the removal of DOC during the multistage A/O phase was 63.55%. The high DOC removal indicated that there was remarkable consumption during the multi-stage A/O phase, which should be verified by the following discussion.

### EEM fluorescence spectra characteristics of DOM

**Fig 3:**
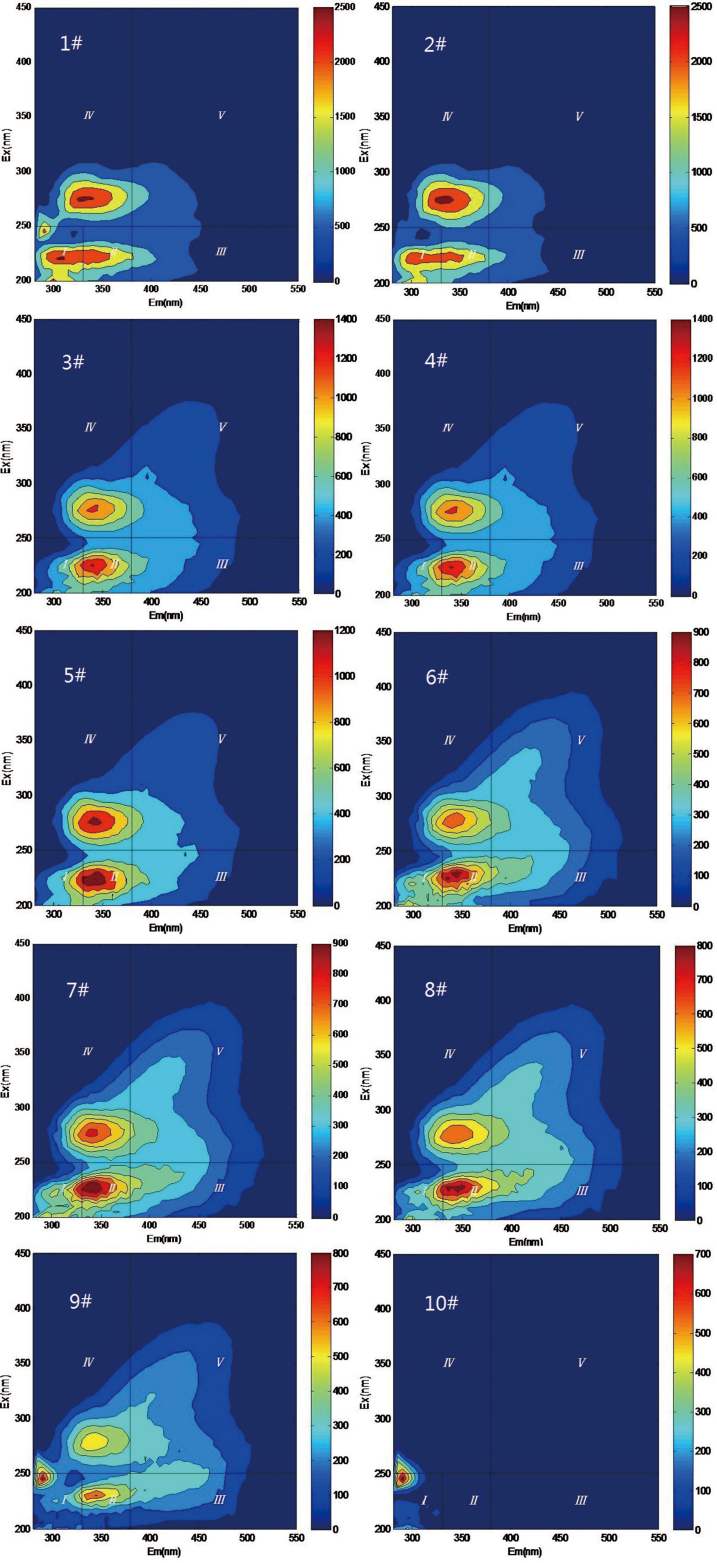
EEM spectra of water samples.

The EEM fluorescence spectra can provide information about the molecular structure and composition of DOM [16]. Four primary fluorescent peaks, i.e., peaks A, B, C and D, were observed in the EEM spectrum of all the samples. Peaks A, B, and C were centered at approximately the Ex/Em of 225/340, 275/340 and 280/400 nm, and they represented tyrosinen-, tryptophan-, and fulvic acid-like substances, respectively [39]. Peaks A and B were attributed to soluble byproduct-like materials of microbial activity [40]. As shown in **Fig 3**, the two peaks disappeared due to the degradation of organic matter as a result of microbial activity. Moreover, peak B showed a blue shift over the entire wastewater reclamation process, and the shifts might be related to a reduction in aromatic structures and a conjugated effect [41]. On the other hand, peak C changed little for the high degree of aromatic polycondensation, which is consistent with previous reports[16], in which it was difficult for the microorganisms to use the fulvic acid-like substances. Peak D (Ex/Em = 325/400 nm) was detected starting at the A^2^O part, which indicated the presence of humic acid-like compounds[42]. The signal became more and more strong thereafter during the multistage A/O process, which is related to the decomposition of dead cells and macromolecular organics [43].

**Fig 4:**
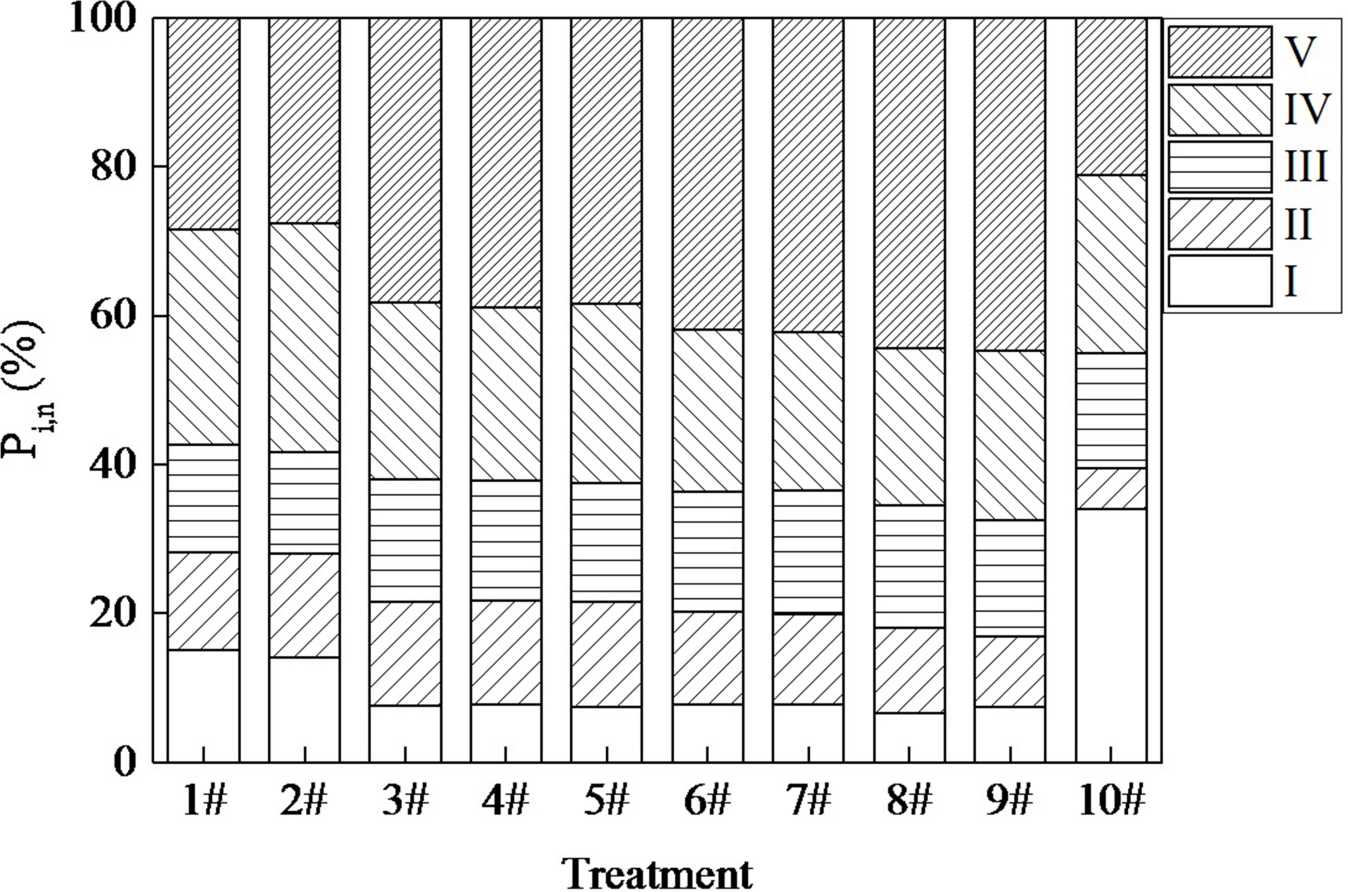
*P*_*i*,*n*_ distributions of EEM spectra.

To better analyze the componential configurations of the DOM, the fluorescence spectra were divided into five zones using the FRI method. The volume integral (Φ_i_) and percent fluorescence responses (*P*_*i*,*n*_) distributions are depicted in **Table 1** and **Fig 4** [19]. As shown in **Table 1**, all the volume integral values of the different areas decreased in accord with the sequence in the Sha-he wastewater reclamation plant, except during the aerobic period. That trend may contribute to the observation of more fluorescent substances under the equal DOC concentration during the aerobic period [43]. The *P*_*h*_*/P*_*p*_ ratios were constantly increasing during the A^2^O and multistage A/O phases (except during the aerobic period in the A^2^O), which indicated that the organic matter was primarily humified [42]. The above results suggest that some easily biodegradable compounds, such as proteins, were continuously consumed, leaving only fulvic- and humic-like substances. It was also noted that the humification of DOM became higher at the multistage A/O phase, indicating that the multistage A/O was more effective for humic- and fulvic-like materials in comparison with the A^2^O technology. In particular, the value of *P*_*h*_*/P*_*p*_ decreased by 61.84% in the MBR systems and the Φ_i_ for Regions II, III, IV, and V decreased more than 90%, whereas the Φ_i_ for Regions I decreased more than 90% from the membrane performance, suggesting that humic-, fulvic- and protein-like matter were removed significantly by the MBR reactor. By contrast, the tyrosine-like materials cannot be removed effectively by the MBR reactor due to their small molecular weights, and they were totally removed by the foregoing biological processing, which is consistent with previous research [44–46].

**Table 1:** Volume integral of different areas in the EEM spectra of DOM.

Region	1#	2#	3#	4#	5#	6#	7#	8#	9#	10#
I	3.57	3.12	1.07	0.91	1.03	0.87	0.84	0.67	0.63	0.35
II	3.13	3.06	1.97	1.62	1.95	1.40	1.31	1.16	0.81	0.05
III	3.44	2.99	2.33	1.90	2.21	1.82	1.79	1.69	1.33	0.16
IV	6.86	6.79	3.35	2.71	3.35	2.45	2.29	2.15	1.94	0.24
V	6.74	6.08	5.42	4.55	5.34	4.72	4.56	4.52	3.81	0.21
*P*_*h*_*/P*_*p*_	0.7507	0.6993	1.21	1.23	1.19	1.39	1.43	1.56	1.52	0.58
TOT	23.76	22.06	14.16	11.72	13.90	11.27	10.82	10.21	8.54	1.04

*P*_*h*_*/P*_*p*_ = ∑ III + V/ ∑ I + II + IV. The ratio of *P*_*i*, *n*_ for the humic- and fulvic-like regions (Regions III and VI) to *P*_*i*, *n*_ for the protein-like regions (Regions I, II, V, and IV) (*P*_*h*_*/P*_*p*_) may hence be used to predict a treatment technique for wastewater.

As shown in **Fig 4**, during the A^2^O and multistage A/O phase, the *P*_*i*,*n*_ values for regions I, II, and IV that were related to protein- and soluble microbial byproduct-like materials decreased by 22.40% and 14.26%, respectively. The *P*_*i*,*n*_ corresponded to region V (humic acid-like substances) increase rapidly during the process. Simultaneously, the predominant remaining compounds were humic-like substances with the *P*_*i*,*n*_ of 44.64%, which was followed by fulvic-like materials with the *P*_*i*,*n*_ of 16.55%. Hudson et al reported that protein-like materials are easily biodegraded with regard to fulvic- and humic-like materials [40]. It was implied that a portion of the aromatic proteins were rapidly biodegraded due to the result of microbial activity during the A^2^O phase, and they were further decomposed during the multistage A/O phase. Therefore, after the disappearance of some easily degradable compounds such as aromatic proteins, the remaining refractory compounds such as fulvic- and humic-like materials accounted for most of the DOM. In comparison with the remarkable increase in humic-like substances, the fulvic acid-like materials changed little. This finding is consistent with previous reports [11], in which the fulvic acid-like materials were first translated into easily degradable organics, and then they might be used as a carbon source for the denitrifying process. Therefore, the fulvic acid-like material may decompose as well in this study.

### Nitrogen removal

**Fig 5:**
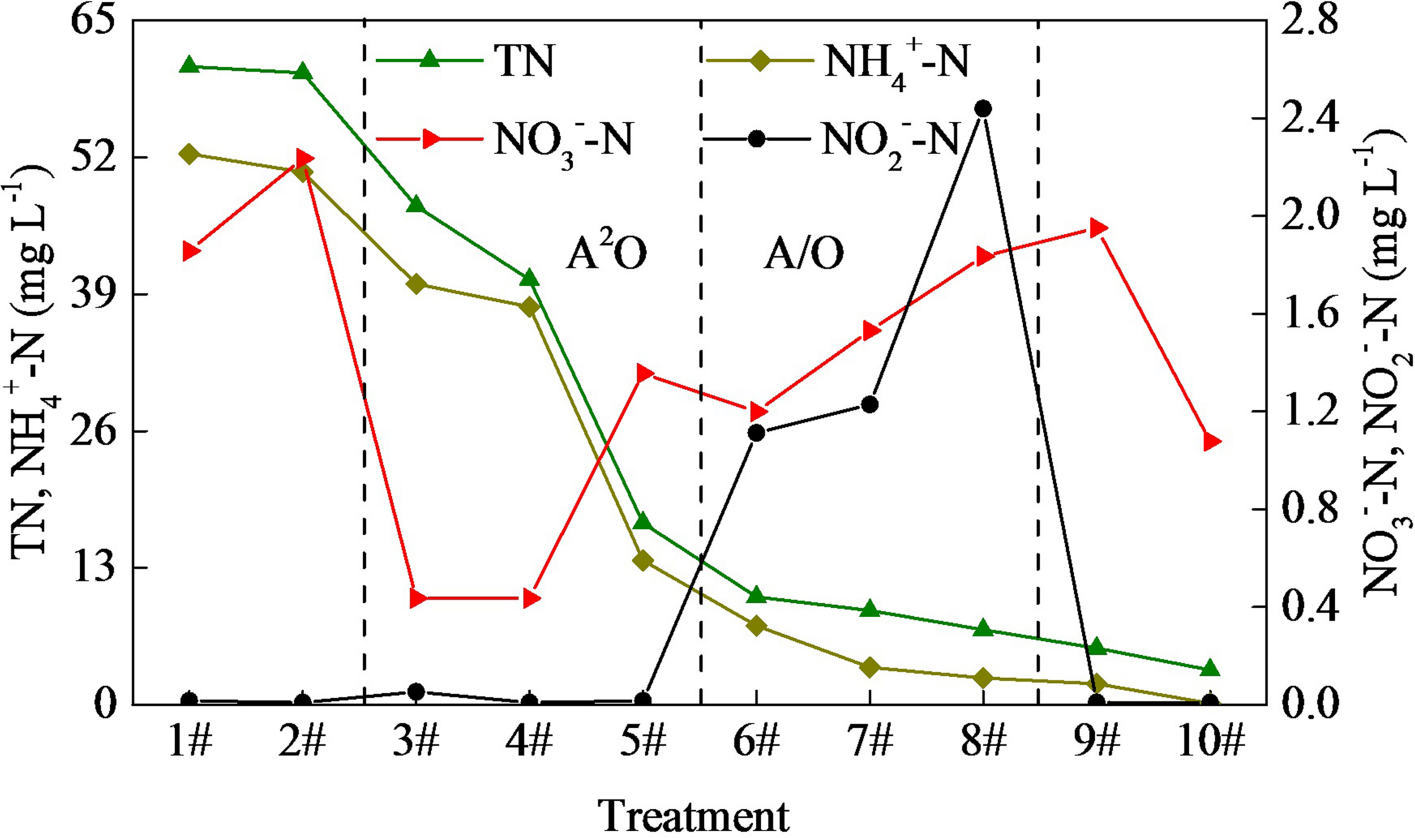
Four forms of nitrogen-changing regularities.

The changes in TN, NH_4_^+^-N, NO_3_^-^-N, and NO_2_^-^-N for each unit are shown in **Fig 5**. In comparison with the TN and NH_4_^+^-N, the NO_3_^-^-N and NO_2_^-^-N showed strong increases with adequate oxygen during the double screen phase, which provided a substrate for the denitrification subsequent reflection. During the A^2^O phase, the effluent TN and NH_4_^+^-N concentrations were 17.25 mg L^-1^ and 13.70 mg L^-1^, and approximately 71.54% of the TN and 73.81% of the NH_4_^+^-N were removed by the anaerobic treatment; the removal of NH_4_^+^-N was lower than the TN removal. Since relatively high organics were present in the raw water, effective heterotrophic denitrification occurred to remove the NO_x_^-^-N during the anaerobic phase. This phenomenon may be attributed to the relatively obvious denitrification reaction and anaerobic ammonium oxidation that occur during the anaerobic period [11, 47]. In addition, the concentrations of TN and NH_4_^+^-N decreased further during the multistage A/O treatment, which showed the multistage A/O process has greater advantage in enhancing the nitrogen removal without adding a carbon source (such as ethanol or acetic acid) at the anoxic stage. The NO_2_^-^-N and NO_3_^-^-N concentrations were steadily increased to 2.44 mg L^-1^ and 1.84 mg L^-1^ during the multistage A/O phase, respectively. In particular, the NO_2_^-^-N concentration, representing the intermediate of the denitrification reaction, increased greatly during the multistage A/O phase, satisfying the requirements for a standard short-range denitrification (a 50% increase) [48]. This phenomenon is consistent with earlier reports regarding advanced nitrogen removal by denitrification, and short-range denitrification may consume organic matter as a carbon source, which would also accelerate the removal of DOC [49–51]. However, the lower organic matter concentration would favor the growth of nitrifying bacteria [52]. It might be another reason for the increasing concentrations of NO_2_^-^-N and NO_3_^-^-N. In conclusion, the results indicated that the multistage A/O treatment exhibited a good performance in relation to nitrogen removal in this wastewater reclamation plant. Finally, a high nitrogen removal efficiency of over 38.42% was observed in the 10# period under the MBR treatment process. The MBR treatment process integrates the advantages of membrane separation technology and bio-treatment technology, and therefore the removal rate of nitrogen will be greatly improved [6].

### Correlation between nitrogen composition variations and the EEM fluorescence volume integral

The Pearson correlations between the volume integral of EEM and the concentration-changing regularities of TN, NH_4_^+^-N, NO_3_^-^-N and NO_2_^-^-N are listed in **Table 2**. The correlation analysis showed that the DOC was significantly positive with the EEM fluorescence volume integral of DOM, which showed that DOC was a suitable parameter for evaluating the DOM content. It was noted that almost every region of the EEM fluorescence volume integral was strongly correlated with the TN and NH_4_^+^-N (except region V), but there was no correlation with the NO_3_^-^-N and NO_2_^-^-N. The correlation coefficient of the TN concentration fluctuates, and the Φ_I,n_, Φ_II,n_, Φ_III,n_, Φ_IV,n_ and Φ_V,n_ were 0.980**, 0.869**, 0.810**, 0.949** and 0.636*, respectively. The order of the correlation was regions I > regions IV > regions II > regions III and V. This result suggests that the content of protein-like and soluble microbial byproduct-like materials was extremely correlated with the nitrogen removal.

**Table 2:** Pearson correlation between different region volumes in EEM spectra and the nitrogen changing regularities (n = 10).

	DOC	TN	NH_4_^+^-N	NO_3_^-^-N	NO_2_^-^-N	Φ_I,n_	Φ_II,n_	Φ_III,n_	Φ_IV,n_	Φ_V,n_	TOT
DOC	1.000	0.985**	0.984**	0.160	-0.336	0.994**	0.927**	0.879**	0.984**	0.725*	0.938**
TN		1.000	0.992**	0.253	-0.288	0.980**	0.869**	0.810**	0.949**	0.636*	0.882**
NH_4_^+^-N			1.000	0.152	-0.397	0.980**	0.882**	0.809**	0.950**	0.623	0.881**
NO_3_^-^-N				1.000	0.763*	0.155	-0.098	-0.083	0.081	-0.094	0.001
NO_2_^-^-N					1.000	-0.354	-0.423	-0.315	-0.341	-0.156	-0.317
Φ_I,n_						1.000	0.901**	0.851**	0.966**	0.683*	0.914*
Φ_II,n_							1.000	0.980**	0.976**	0.897**	0.991**
Φ_III,n_								1.000	0.943**	0.958**	0.988**
Φ_IV,n_									1.000	0.827**	0.982**
Φ_V,n_										1.000	0.916**
TOT											1.000

*.Correlation is significant at the 0.05 level (2-tailed).

**. Correlation is significant at the 0.01 level (2-tailed).

As shown in **Table 2**, the TN not only exhibits a clear positive correlation with the Φ_n_ value of fulvic acid-like materials, but it also correlates positively with the Φ_n_ value for humic acid-like materials. The DOM in regions III and V only represent the fluorescence information on fulvic-like and humic-like substances. Combined with the advantage of nitrogen removal in the back-end A/O and the lack of carbon source, it can be concluded that nitrogen removal may be achieved with both the fulvic-like and humic-like substances when there are less protein-like and soluble microbial byproduct-like materials in the DOM, which were usually considered as organics that are difficult for microorganisms to use. Moreover, the TN removal was more significantly correlated with fulvic acid-like materials (R^2^ = 0.810, P < 0.01) than humic acid-like materials (R^2^ = 0.636, P < 0.05). Compared with the fulvic acid-like substances, humic acid-like substances were much more difficult to use as carbon sources during the nitrogen removal process.

## Conclusions

This study was conducted to investigate the relationship between nitrogen removal and the DOM in the Sha-he wastewater reclamation plant. The effluent water quality of the biological treatment reached the first grade A standard of “Cities sewage treatment plant pollutant discharge standard” (GB18918-2002). The effluent of the membrane bioreactor reached the “Standards of reclaimed water quality” (SL368-2006). Moreover, the multistage A/O technology can effectively improve the nitrogen removal ability of wastewater reclamation plant, and the NO_2_^-^-N accumulated to more than 50 percent during the multistage portion of this phase, satisfying the requirements of standard short-range denitrification. In particular, the higher humification of organic matter indicated that the multistage A/O was more effective than the A^2^O technology as analyzed by FRI method, with both humic acid-like materials and fulvic-like substances being found in the DOM during the TN removal process. Compared with the fulvic acid-like substances, the humic acid-like substances were much more difficult to use as a carbon source during the nitrogen removal process. Overall, our results could improve understanding about the role of DOM in nitrogen removal and thus provide aid in developing technological improvement in the wastewater reclamation plant.

## Supporting information

S1 Table Variations in the COD and DOC. (Data for **Fig 2**)
(DOC)

S2 Table *P*_*i*,*n*_ distributions of EEM spectra. (Data for **Fig 4**)
(DOC)

S3 Table Four forms of nitrogen-changing regularities. (Data for **Fig 5**)
(DOC)
