# Supplementary material for: Spectroscopic characterization of DOM and the nitrogen removal mechanism during wastewater reclamation plant
Source: PLoS One. 2017 Nov 17;12(11):e0187355. doi: 10.1371/journal.pone.0187355 (PMC5693440; doi:10.1371/journal.pone.0187355)
Supplement: S1 Table — (Data for Fig 2) (DOC) [file pone.0187355.s001.doc]

**S1 Table.** Variations in the COD and DOC. (Data for **Fig. 2**)

|  | DOC | COD |
| --- | --- | --- |
| 1# | 30.98 | 274.97 |
| 2# | 28.94 | 238.68 |
| 3# | 15.34 | 149.57 |
| 4# | 7.53 | 110.39 |
| 5# | 9.22 | 105.97 |
| 6# | 7.02 | 75.31 |
| 7# | 6.45 | 68.84 |
| 8# | 5.96 | 48.57 |
| 9# | 5.69 | 34.97 |
| 10# | 3.19 | 13.36 |
