# Supplementary material for: Spectroscopic characterization of DOM and the nitrogen removal mechanism during wastewater reclamation plant
Source: PLoS One. 2017 Nov 17;12(11):e0187355. doi: 10.1371/journal.pone.0187355 (PMC5693440; doi:10.1371/journal.pone.0187355)
Supplement: S2 Table — (Data for Fig 4) (DOC) [file pone.0187355.s002.doc]

S2 Table. *P _i,n_* distributions of EEM spectra. (Data for Fig. 4)

|  | **Region I** | **Region II** | **Region III** | **Region IV** | **Region V** |
| --- | --- | --- | --- | --- | --- |
| 1# | 15.03 | 13.21 | 14.48 | 28.91 | 28.38 |
| 2# | 14.17 | 13.88 | 13.56 | 30.78 | 27.60 |
| 3# | 7.60 | 13.95 | 16.50 | 23.69 | 38.26 |
| 4# | 7.77 | 13.90 | 16.25 | 23.20 | 38.88 |
| 5# | 7.47 | 14.03 | 15.96 | 24.15 | 38.40 |
| 6# | 7.78 | 12.42 | 16.18 | 21.74 | 41.87 |
| 7# | 7.81 | 12.18 | 16.57 | 21.25 | 42.19 |
| 8# | 6.59 | 11.44 | 16.55 | 21.11 | 44.30 |
| 9# | 7.45 | 9.50 | 15.65 | 22.76 | 44.64 |
| 10# | 34.03 | 5.41 | 15.52 | 23.92 | 21.11 |

*P_i,n_*, percent fluorescence responses distributions of different areas in the EEM spectra of DOM (%).
