# Supplementary material for: Spectroscopic characterization of DOM and the nitrogen removal mechanism during wastewater reclamation plant
Source: PLoS One. 2017 Nov 17;12(11):e0187355. doi: 10.1371/journal.pone.0187355 (PMC5693440; doi:10.1371/journal.pone.0187355)
Supplement: S3 Table — (Data for Fig 5) (DOC) [file pone.0187355.s003.doc]

**S3 Table.** Four forms of nitrogen-changing regularities. (Data for **Fig. 5**)

|  | TN | NH_4_^+^-N | NO_3_^-^-N | NO_2_^-^-N |
| --- | --- | --- | --- | --- |
| 1# | 60.63 | 52.33 | 1.86 | 0.02 |
| 2# | 60.01 | 50.61 | 2.24 | 0.01 |
| 3# | 47.37 | 39.97 | 0.44 | 0.05 |
| 4# | 40.40 | 37.81 | 0.44 | 0.01 |
| 5# | 17.26 | 13.71 | 1.36 | 0.02 |
| 6# | 10.24 | 7.51 | 1.20 | 1.11 |
| 7# | 8.95 | 3.56 | 1.53 | 1.23 |
| 8# | 7.12 | 2.54 | 1.84 | 2.44 |
| 9# | 5.38 | 1.99 | 1.95 | 0.01 |
| 10# | 3.31 | 0.14 | 1.08 | 0.01 |
